# Longitudinally extensive transverse myelitis with mycobacterium tuberculosis infection

**DOI:** 10.1007/s13760-021-01723-0

**Published:** 2021-07-05

**Authors:** Le Fang, Yushuang Gong, Kai Han, Yalin Lv, Miao Li, Jie Wang

**Affiliations:** 1 Department of Neurology, The China-Japan Union Hospital of Jilin University, Changchun, 130031 China; 2 Department of Neurosurgery, The China-Japan Union Hospital of Jilin University, Changchun, 130031 China

Dear Editor,

Longitudinal extensive transverse myelitis (LETM) is defined as a spinal cord lesion that extends over three or more vertebrae, as seen on MRI of the spine [1]. LETM is a common characteristic feature of neuromyelitis optica spectrum disease (NMOSD) or various autoimmune diseases [2]. While in some cases, the common antibodies are negative and related to some hidden and unknown causes. We report a vegetarian woman presented sudden onset of quadriplegia with LETM in cervical and thoracic spinal cord which was confirmed with neuroimaging. On MRI, the patient was found exhibit atypical longitudinal extensive transverse abnormal signals that mimicked NMOSD. We tracked for possible reasons that may be related to LETM and found encranial and pulmonary *Mycobacterium* tuberculosis (MTB) infection. Systemic screening indicated there was no evidence supporting autoimmune diseases such as NMOSD or paraneoplastic neurological syndrome (PNS). The patient showed good clinical response to anti-tuberculosis therapy, corticosteroids and immunoglobin.

Usually diagnosis of LETM from NMOSD or tracking for autoimmune reasons of LETM is difficult. Our case highlights that MTB infection might be an important but overlooked cause of LETM. Symptoms of MTB are usually nonspecific, as seen in the current case, and the duration of symptoms before diagnosis can vary [3].

## Case report

A 45-year-old woman of Han Nationality was admitted to our hospital in April 2018. She developed rapidly progressive paraparesis, sensory disturbances, bladder/bowel dysfunction and dyspnea. The first onset symptom was numbness in both lower limbs then quickly developed to paraparesis and dyspnea. The patient was hospitalized to neurointensive care unit to receive close observation and treatment. The patient showed paraparesis, sensory disturbances, bladder/bowel dysfunction and dyspnea, as while as hysphagia, Horner’s syndrome and low-grade fever. The patient had palpitations, chest tightness, constipation, like Horner’s syndrome, were certain extent related to dysautonomia. She denied the apparent pain in the back or chest. She denied any history of infection, tumor or contact of special medcines.

Physical examination: The patient had normal consciousness, normal blood pressure, increasing heart rate (100–110/min) and harsh respiration. The body temperature was 37.0–38.0 °C. She complained mild dyspnea and some chest tightness. There was Horner’s syndrome in the left side. The bilateral lower limbs power was 0/5 and bilateral upper limbs power was 3/5 with absent reflexes in all limbs. The abdominal reflexes were absent. The sensory level was identified at T2 and the deep and shallow senses below T2 level obviously declined. The pyramidal signs of Babinski’s were positive in both sides. There was no skin rash and no lymphadenectasis. The patient got acute urinary retention soon after hospitalization and received indwelling catheter.

Laboratory findings: peripheral blood examinations: RBC 4.21 × 10^12^/L, WBC 8.38 × 10^9^/L, neutrophilic granulocyte 82.4%, lymphocyte 12.2%, hemoglobin (HG) 132.0 g/L. Blood glucose 5.86 mmol/L, renal and liver functions were normal. Erythrocyte sedimentation rate (ESR) was 20 mm/h. Serum markers for autoimmune and connective tissues (ANA, Anti-dsDNA, Anti-nucleosome, Anti-histones, Anti-Sm, Anti SS-A, Anti RO, Anti Scl-70, Anti Rib-P-Protein, Anti-JO, Anti-SS-B) were negative. Immunoglobulin IgG, IgA, IgM, C3, C4 were normal. Blood culture was negative. Serology results for tubercle bacillus, rubella and Epstein–Barr virus, Lyme and brucella were negative. Serum viral serology for hepatitis B virus (HBV), hepatitis C virus (HCV) and human immunodeficiency virus were unremarkable. Antiphospholipids antibody (APLA) was negative. Skin tuberculin sensitivity test was positive. Polymerase chain reaction (PCR) for MTB on three consequtive sputum samples was positive.

The lumbar puncture revealed intracranial pressure (ICP) was 20cmH_2_O (1cmH_2_O = 9.8 kPa). Cerebrospinal fluid (CSF) examinations: CSF protein 28 mg/dL, WBC 0, glucose 86.4 mg/dL, chloridum 431.4 mg/dL, NMO/AQP4 antibodies, MOG antibodies, myelin basal protein (MBP) and oligoclonal bands (OB) detection were absent in serum and CSF. CSF culture for bacterium was negative. PCR for MTB in CSF was positive while PCR for herpes simplex virus was negative. CSF acid-fast bacillus (AFB) stain, and an agglutination test for brucella were negative.

**Fig. 1:**
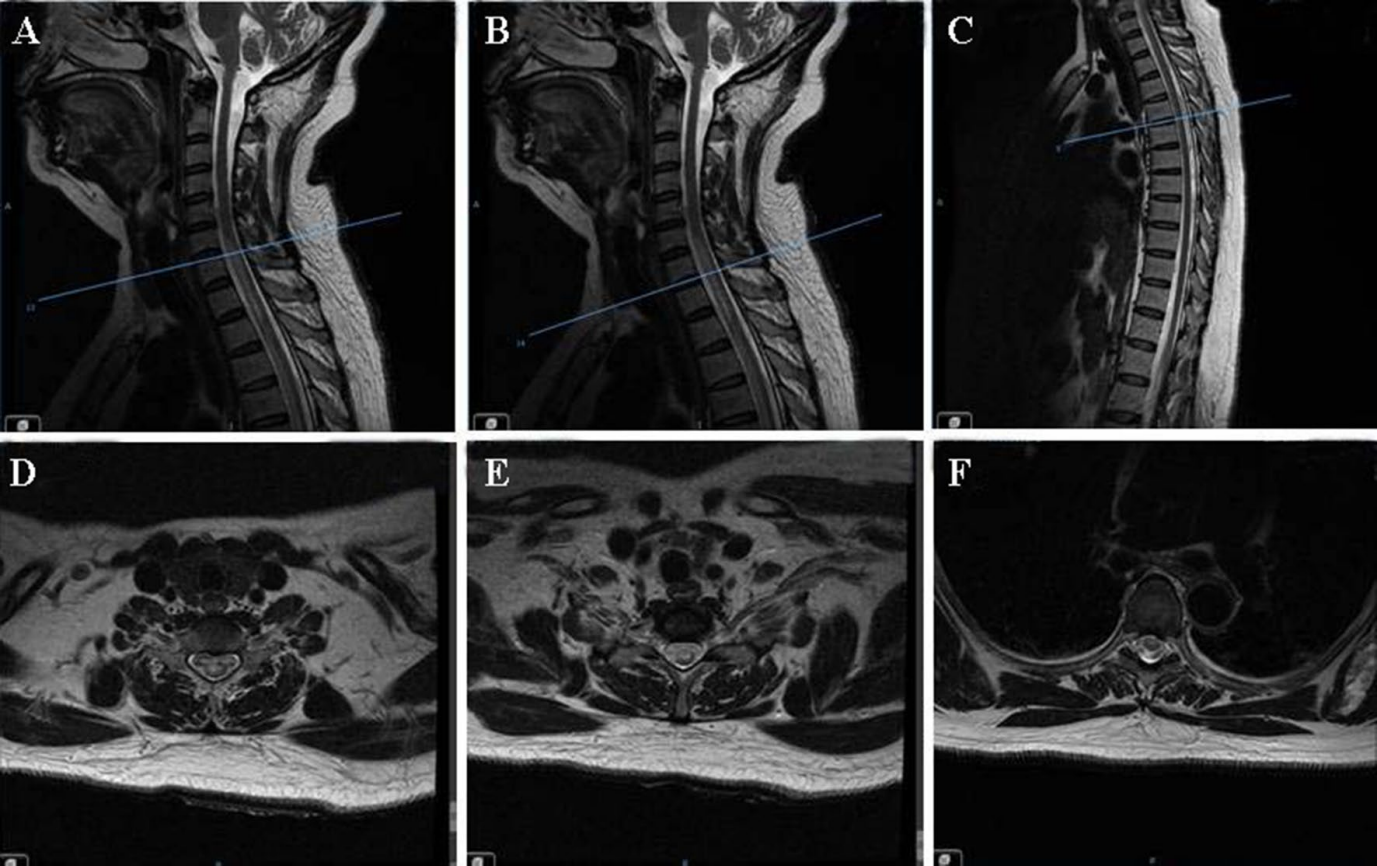
Magnetic resonance imaging (MRI) obtained on the third day of onset of the disease (sagittal section **A**–**C**; transverse section corresponding to the scaleplate respectively **D**–**F**). T2 hyperintense linear mass best appreciated on sagittal sections, showing long segment myelitis, extending from C5–T2

Images: magnetic resonance imaging (MRI) showed hyperintens best appreciated on T-2-weighted sections, showing long segment myelitis, extending from C5–T2 (Fig. 1). The pulmonary CT scan suggested active pulmonary tuberculosis in bilateral upper lobes.

**Fig. 2:**
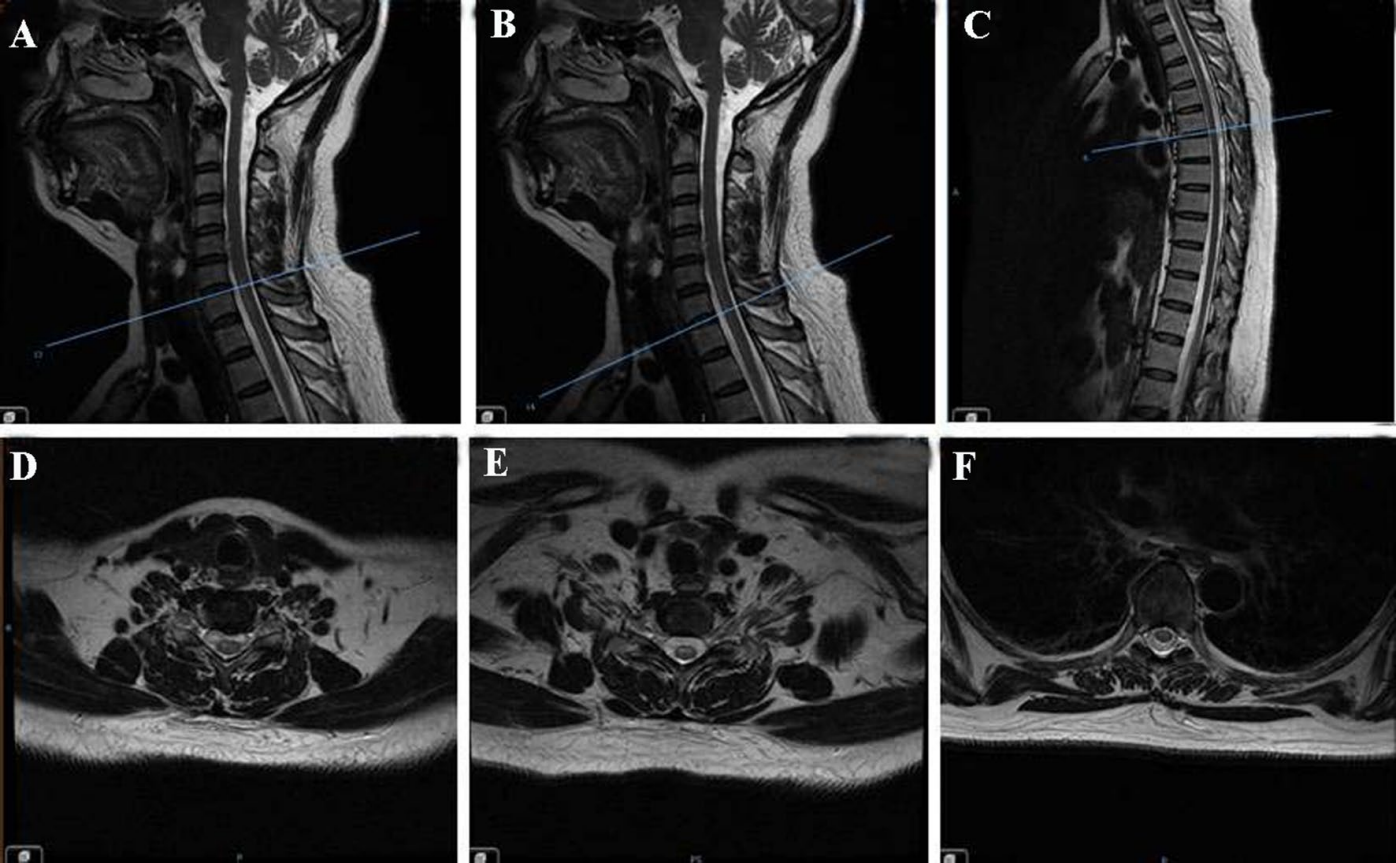
Magnetic resonance imaging (MRI) obtained one month of onset of the disease (sagittal section **A**–**C**; transverse section corresponding to the scaleplate, respectively **D**–**F**). The lesions were significantly absorbed compared with the anterior imaging

The clinical and imaging findings supported a diagnosis of LETM and pulmonary tuberculosis. The differential diagnosis required spinal cord ischemia as its acute onset. As the patient had no obvious neuroroot pain and the longitudinal extensive lesions of cervical and thoracic vetebrae did not meet the anatomical vascular supply, which was not consistent with the characteristics of ischemia. Furthermore, the patient was a 45-year-old woman with no risk factors for vascular disease, such as hypertension or diabetes. LETM and pulmonary tuberculosis were finally diagnosed. The patient received high-dose and short-period steroid therapy (500 mg of methylprednisolone intravenously per day for five continuous days, then halve the dose and stop it after 2 days) and intravenous immunoglobin (400 mg/kg per day for five continuous days). At the same time, the patient received sufficient anti-tuberculosis therapy. The patient showed satisfied recovery and was afebrile without chest tightness or dysphagia. Muscle strength of the extremities gradually recovered. On 1 month follow-up, the bilateral lower limbs power was 3/5 and bilateral upper limbs power was 4/5. The patient was able to stand with part of support. She still complained of occasional urinary incontinence. MRI obtained 1 month after the onset of the disease showed the lesions were significantly absorbed compared with the anterior imaging (Fig. 2).

## Discussion

LETM often indicates initiating systemic immune responses for known or unknown reasons, which is different from short extensive myelitis. Typically LETM is associated with NMOSD with positive serum NMO/AQP4 antibodies [2]. However, not all LETM are NMOSD [4, 5]. Hyun JW followed up 108 patients with LETM for at least 2 years and found nearly half of cases were idiopathic aquaporin-4 antibody negative LETM with different features, which could not be simply diagnosed as NMOSD. When aquaporin-4 antibody is negative, it is necessary to be very careful in diagnosis of NMOSD [2, 5]. LETM can occur in various autoimmune diseases, such as multiple sclerosis (MS), systemic lupus erythematosus (SLE) or Sjögren syndrome, or follow infectious diseases, such as tuberculosis infection and HIV infection [6–8]. There is view that MTB may share antigens with myelin basic protein although no antibodies are found so far [8]. The possible infectious agents such as MTB are worth further discussing or exploring. Recently report reveal transverse myelitis can also occur following SARS-CoV-2 infection [9]. The pathogenesis of LETM is unclear so far and few correlative factors are reported or studied.

We report a patient developed LETM in cervical and thoracic spinal cord with possible antigenicity related to MTB. Diagnosis of LETM was made according to the clinical manifestation and radiological data. We tracked for possible reasons that may be related to LETM, while there was no evidence supporting autoimmune diseases such as NMOSD, PNS or other infection except MTB. Laboratory findings indicated conceivable intracranial MTB infection and active pulmonary tuberculosis. Although initial investigations were equivocal for an infectious etiology, subsequent investigations led to a diagnosis of LETM with MTB infection. PCR for MTB in the CSF is a very specific test with high sensitivity, which suggests acute or previous intracranial MTB infection [10]. Tubercular etiology and its associated immune response were suggested in our case in view of positive TB-PCR in the CSF and pulmonary tuberculosis.

Association between LETM and MTB is a very worthy of discussion. The pathogenesis of LETM remained unclear science the absence of pathological data and failed to find any specific antibody. Associations between NMOSD and pulmonary tuberculosis have been suggested by a number of case reports and series, the most likely mechanism being immune-mediated inflammatory demyelination of the optic nerves and spinal cord triggered by infection of MTB [8, 11, 12]. Li R investigated neuromyelitis optica and tuberculosis in a Chinese population and put forward the view that MTB may shares antigens with myelin basic protein (MBP) [8]. Sahu reported four cases of LETM with mycobacterium tuberculosis and highlight the fact that MTB infection might be more common than usually suspected in LETM [13]. Usually LETM is difficulty to make etiological diagnosis and needs to be distinguished from NMOSD or abscesses [14].

Based on our belief that the immune injury was the main pathological process, short-period corticosteroids and immunoglobin were administrated. The patient showed good clinical response to anti-tuberculosis therapy, corticosteroids and immunoglobin. Corticosteroids are not absolute controversial in the treatment of central nervous system tuberculosis [15]. We believe that short-period corticosteroids may be adjuvant to antituberculous treatment. Although pathological data were not available in this case, we hypothesized that MTB shares antigens with myelin basic protein, thus sensitizing lymphocytes to mycobacterium and facilitating their attachment to myelin. LETM may develop by possible antigenicity from MTB. The antigenicity need to rely on further experimental confirmation, especially base on establishment of reliable animal models.

Our case highlights the fact that MTB might be an important but overlooked cause of LETM. LETM should be diagnosed considerately from NMOSD and searching for relevant factors is needed. Prompt identification of underlying etiology by contrast examination and systemic survey is cruci al for the patient assumed as LETM.
